# *XPO5* Polymorphism in Colon Cancer Patients: A Cross-Sectional Study

**DOI:** 10.3390/ijms27010345

**Published:** 2025-12-29

**Authors:** Tugba Agbektas, Husnu Cagrı Genc, Cemile Zontul, Ayca Tas

**Affiliations:** 1 Department of Food Processing Technologies Services, Yıldızeli Vocational School, Sivas Cumhuriyet University, Sivas 58140, Türkiye; tubaagbektas@cumhuriyet.edu.tr; 2 Department of General Surgery, Faculty of Medicine, Sivas Cumhuriyet University, Sivas 58140, Türkiye; cagrigenc@cumhuriyet.edu.tr; 3 Department of Chemistry and Chemical Processing Technologies Services, Yıldızeli Vocational School, Sivas Cumhuriyet University, Sivas 58140, Türkiye; cemilezontul@cumhuriyet.edu.tr; 4 Department of Medical Biochemistry, Faculty of Medicine, Sivas Cumhuriyet University, Sivas 58140, Türkiye

**Keywords:** *XPO5* gene, colon cancer, rs11544382, polymorphism

## Abstract

(1) This cross-sectional study aims to elucidate the association between the *XPO5* gene polymorphism (rs11544382) and colon cancer (CC). (2) Genotyping of *XPO5* (rs11544382) was performed in 120 individuals (60 CC patients and 60 controls) using real-time PCR (qPCR). Logistic regression and Chi-square (χ^2^) tests were used for statistical analysis. (3) Evaluation of the *XPO5* gene polymorphism in CC and control groups revealed no statistically significant association between the mutant (GG) genotype and either the wild-type (AA) or heterozygous (AG) genotypes (χ^2^ = 2.07, *p* = 0.151). The AG genotype was predominant in both patients (86.7%) and controls (91.7%). Smoking and alcohol consumption showed significant associations with CC (*p* < 0.05). Although the rs11544382 polymorphism was not associated with CC risk, this is a cross-sectional study. In light of these findings, larger and more comprehensive studies with increased sample size are required to clarify the relationship between the *XPO5* gene polymorphism (rs11544382) and CC.

## 1. Introduction

CC is the third most commonly diagnosed malignancy worldwide and the second leading cause of cancer-related mortality, with incidence rates continuing to rise globally [1,2]. It predominantly affects older adults, although it may occur at any age, and typically originates from the formation of small clusters of abnormal cells, known as polyps, within the colonic epithelium. Over time, these polyps may undergo malignant transformation and progress into invasive carcinoma [3]. According to projections by the International Agency for Research on Cancer (IARC), the global incidence of colorectal cancer is expected to rise by approximately 56% between 2020 and 2040, exceeding 3 million new cases annually. During the same period, mortality is projected to increase by 69%, reaching nearly 1.6 million deaths by 2040. This sharp rise will be particularly pronounced in populations with a high Human Development Index [4,5]. Early detection and removal of premalignant adenomas can substantially reduce disease incidence, with surgical excision remaining the cornerstone of curative treatment, particularly in early-stage disease, and being associated with favorable outcomes [6,7]. Recurrence risk is influenced by nodal involvement, yet overall, surgical resection is considered highly effective in the management of localized tumors [8]. Conventional diagnostic methods, such as fecal occult blood testing (FOBT), are limited by low sensitivity and specificity, dietary restrictions, and relatively high costs. These drawbacks underscore the need for non-invasive, cost-effective, and highly sensitive screening strategies. In this context, approaches based on microRNA (miRNA) profiling and proteomic transcriptomic assays have gained increasing attention [9]. miRNAs are endogenous, small non-coding RNAs approximately 22 nucleotides in length, and their biogenesis involves a multistep process. Initially, RNA polymerase II (RNAPII) transcribes pri-miRNAs containing imperfect hairpin structures. These are subsequently cleaved into 70 nucleotide pre-miRNAs, which are exported to the cytoplasm via the RanGTP/exportin-5 (*XPO5*) complex. In the cytoplasm, pre-miRNAs are processed into mature miRNAs, which then participate in gene regulation either by inducing mRNA degradation or by repressing translation through the RNA-induced silencing complex (RISC) [10]. Emerging evidence indicates that miRNA expression is frequently downregulated in tumor tissues, potentially reflecting defects in the biogenesis machinery [11,12]. Among the proteins essential for this process, *XPO5* has received particular attention. During the preparatory phase of the cell cycle, *XPO5* is upregulated through PI3K-dependent post-transcriptional mechanisms, thereby enhancing global miRNA expression. Inhibition of *XPO5* induction disrupts normal proliferation and delays the G1/S transition, underscoring its central role in cell–cycle regulation [13].

Both increased and decreased expression levels of *XPO5* have been observed across different cancer types. Overexpression of *XPO5* has been reported in colorectal, breast, bladder, and thyroid carcinomas, as well as in melanoma [14,15]. Although the molecular mechanisms underlying *XPO5* overexpression and its associated tumorigenic activity remain unclear, amplification of *XPO5* resulting from chromosome 6p polyploidy has been shown to be associated with gastric cancer [16]. In contrast, reduced *XPO5* expression levels, attributed to the recurrent single nucleotide polymorphism rs11077 located in the 3′ untranslated region (3′UTR) of the *XPO5* gene, have been closely associated with thyroid, liver, laryngeal, and colorectal cancers, as well as leukoplakia [17,18]. In line with these observations, recent studies further suggest that epigenetic alterations affecting *XPO5*-mediated pathways contribute to tumorigenesis, and deregulated microRNA (miRNA) expression has been recognized as a hallmark of colorectal cancer (CRC) and several other human malignancies [9]. Updated mechanistic models emphasize Exportin-5–mediated nuclear export as a rate-limiting step in miRNA maturation [19], while emerging evidence indicates that *XPO5* also facilitates pri-miRNA processing beyond its canonical export function [20]. Consequently, dysregulation or mutation of *XPO5* has been linked to impaired miRNA maturation and altered oncogenic signaling pathways [21]. Within this context, Leaderer et al. investigated the positions of the rs11544382 and rs34324334 polymorphisms within the predicted functional domains of the *XPO5* protein and reported that rs11544382 is located in the Pfam-B PB001127 region, whereas rs34324334 resides within an Exportin-1/Importin-β-like domain. These findings suggest that both polymorphisms may have occurred within functionally conserved regions of the protein. Furthermore, Leaderer et al. demonstrated that the amino acid substitution associated with rs11544382 was consistently predicted to be deleterious by multiple in silico tools, including SIFT, PolyPhen, SNPs3D, and Pmut, suggesting that this polymorphism may alter the structural integrity and, consequently, the function of the *XPO5* protein [22].

The aim of this study is to investigate the association between the *XPO5* gene polymorphism (rs11544382) and CC in a Turkish cohort using qPCR.

## 2. Results

### 2.1. Patient and Control Group Findings

The demographic characteristics of the study population are summarized in Table 1. Among the control group, 49 (48.0%) participants were male and 11 (61.1%) were female, whereas the CC group consisted of 53 (52.0%) males and 7 (38.9%) females. No statistically significant difference in gender distribution was observed between cases and controls (*p* = 0.306). Gender was not significantly associated with CC risk in univariate binary logistic regression analysis (OR = 1.70, *p* = 0.31). Based on the median age values, the median age of male patients was 60 years (range: 46–85), while the median age of male controls was 64 years (range: 34–76), with no statistically significant difference between the groups (*p* = 0.237). A similar pattern was observed for females, where the difference in median age approached but did not reach statistical significance (*p* = 0.053). Regarding smoking history, 13 (43.3%) males and 1 (3.3%) female patient in the CC group were smokers, compared to 33 (67.3%) males and 1 (9.1%) female in the control group. With respect to alcohol consumption, 10 (33.3%) males and 3 (10.0%) females in the CC group reported alcohol use, while in the control group, only 4 (8.2%) males consumed alcohol and no females reported alcohol use. A family history of cancer was present in 8 (26.6%) of the CC patients and 10 (48.6%) of the controls. However, no statistically significant difference was observed between the groups (*p* > 0.05) (Table 1).

### 2.2. Genotypic and Allelic Association Analysis of XPO5 rs11544382 in CC and Control Groups

Allelic distributions of the *XPO5* rs11544382 polymorphism in CC patients and controls are summarized in Table 2. The frequency of the A allele was 56.6% in the CC group and 54.1% among controls, whereas the G allele was present in 43.4% and 45.9% of the groups, respectively. Statistical comparison using the Chi-square test showed no significant difference in allele frequencies between the two groups (χ^2^ = 0.15, *p* = 0.696), indicating that the rs11544382 alleles are similarly distributed in CC patients and healthy individuals. The crude odds ratio supported this finding, as the A allele did not confer an increased or decreased risk of CC compared with the G allele (OR = 0.90, 95% CI: 0.53–1.55).

The genotypic distributions of the *XPO5* rs11544382 polymorphism in CC patients and controls are summarized in Table 3. In the control group, the frequencies of the AA, AG, and GG genotypes were 8.3%, 91.7%, and 0.0%, respectively, while CC patients exhibited AA, AG, and GG genotype frequencies of 13.3%, 86.7%, and 0.0%. The marked predominance of the heterozygous AG genotype and the complete absence of the GG genotype were observed in both groups. Genotype-based comparisons did not reveal a statistically significant difference between CC patients and controls. Consistently, the crude odds ratio for rs11544382 was 0.59 (95% CI: 0.18–1.92), indicating no measurable association between genotype distribution and CC risk. Hardy–Weinberg equilibrium analysis using an exact test demonstrated a significant deviation from equilibrium in both the control and patient groups, reflecting a highly unbalanced genotype distribution at this locus.

### 2.3. Association of CC with Smoking, Alcohol Consumption, and Family History of Cancer

The multivariable logistic regression analysis evaluating clinical and genetic risk factors for CC is presented in Table 4. The *XPO5* rs11544382 genotype did not demonstrate a significant association with CC risk. Both the crude OR (0.59; 95% CI: 0.18–1.92) and the adjusted OR (0.62; 95% CI: 0.19–2.01) indicated no meaningful increase or decrease in disease susceptibility, consistent with the non-significant *p* = 0.41. These findings confirm that rs11544382 is not an independent predictor of CC in this cohort. In contrast, smoking status emerged as a significant risk factor. Individuals with a smoking history had a markedly higher likelihood of developing CC, with a crude OR of 3.84 (95% CI: 1.72–8.55). This association remained significant after adjusting for potential confounders, with an adjusted OR of 3.12 (95% CI: 1.25–7.76; *p* = 0.014), indicating that smoking independently contributes to CC risk. Alcohol consumption was also significantly associated with CC. The crude OR (3.87; 95% CI: 1.18–12.67) suggested an elevated risk among alcohol users, and this association persisted in the adjusted model (adjusted OR = 2.93; 95% CI: 1.03–9.24; *p* = 0.042). These results suggest that alcohol use may serve as an independent lifestyle-related risk factor for colon cancer. Conversely, family history of cancer did not show a significant relationship with CC development. Both the crude OR (0.76; 95% CI: 0.28–2.10) and the adjusted OR (0.49; 95% CI: 0.14–1.64) indicated no evidence of increased risk, which was supported by the non-significant *p* = 0.22. These results show that, within this sample, family history is not a determining factor for CC susceptibility.

## 3. Discussion

This study investigated the potential contribution of the *XPO5* rs11544382 polymorphism to CC susceptibility. Our genotyping results showed no significant differences in allele or genotype frequencies between CC patients and controls, suggesting that rs11544382 is unlikely to influence CC risk in the studied population.

However, the genotype distribution of rs11544382 deviated markedly from Hardy–Weinberg equilibrium in both the control and patient groups, mainly due to a pronounced excess of heterozygous individuals and the complete absence of the GG genotype. Based on the observed allele frequencies, approximately 11–13 GG homozygotes would be expected in each group under HWE conditions. Such an atypical and highly unbalanced genotype distribution may reflect methodological or population-specific factors and substantially limits the effective genetic variability at this locus, thereby reducing the statistical power to detect a true genotype–phenotype association. Therefore, the lack of association observed in this study should be interpreted with appropriate caution.

Similarly, a study conducted in Korean women with primary ovarian insufficiency reported that rs11544382 was monomorphic, supporting the notion that population-specific genetic structures may constrain the detectability of this variant’s effects. Collectively, these findings indicate that rs11544382 does not exert a measurable germline effect on CC susceptibility [23]. Consistent with this, our study did not identify an association between rs11544382 and cancer risk. Unlike the Korean cohort, however, the heterozygous AG genotype was predominant among both CC patients and controls in our population, further supporting the absence of differential genotype distribution. The lack of the GG genotype in all participants suggests that the homozygous form of the G allele is extremely rare in this population and may have influenced the statistical outcomes. Previous research has shown that *XPO5* polymorphisms may vary across cancer types and populations [24,25]. Consistent with these findings, growing evidence indicates the presence of multiple single nucleotide polymorphisms (SNPs) within the *XPO5* gene. Among these, two missense SNPs rs11544382 (M1115T) and rs34324334 (S2411N) have been identified in the *XPO5* coding region. Notably, variant genotypes of rs11544382 have been reported to be significantly associated with an increased risk of breast cancer compared with the common homozygous genotype [22]. In addition, rs11077 (GRCh38.p7, chr6:43523209), a potential microRNA-associated SNP (miR-SNP) located in the 3′ untranslated region (3′UTR) of the *XPO5* gene, has been extensively investigated across various malignancies. For example, the rs11077 AC genotype has been associated with a more favorable chemotherapeutic response in patients with metastatic colon cancer [26]. Furthermore, the AC + CC genotypes of rs11077 have been reported to be significantly associated with improved survival and reduced recurrence rates in patients with non-small cell lung cancer (NSCLC) [27]. In particular, the more common heterozygous AC genotype has been linked to a better chemotherapeutic response in NSCLC, as well as prolonged overall survival in patients with chemotherapy-sensitive multiple myeloma and NSCLC [28,29]. However, it should be noted that the AC genotype has also been associated with an increased risk of esophageal and renal cell carcinomas, suggesting that the functional impact of rs11077 may vary depending on cancer type and biological context [30,31]. For example, a case–control study in Chinese individuals demonstrated that rs11077 was significantly associated with thyroid cancer risk, with the G allele linked to reduced *XPO5* expression in tumor tissues [17]. Such evidence suggests that genomic mutations, aberrant transcription, or other regulatory mechanisms may substantially affect *XPO5* protein levels [32]. Although rs11544382 does not alter the amino acid sequence of *XPO5*, its potential regulatory effects cannot be excluded. As a non-coding variant [10], any functional relevance would more likely arise from modulation of transcriptional activity or mRNA processing rather than structural changes in the protein. The significant associations observed for smoking and alcohol consumption in our study raise the possibility that environmental exposures interact with subtle regulatory variants influencing microRNA processing pathways or oxidative stress–related mechanisms in which *XPO5* plays a role. Although such interactions remain speculative, they provide a biologically plausible framework for future mechanistic investigations. In breast cancer, rs11544382 has been associated with increased susceptibility, particularly among Caucasian populations, and hypermethylation of the *XPO5* promoter has been linked to reduced cancer risk [22]. Moreover, the rs11544382 (A>G) polymorphism has been reported to significantly increase breast cancer risk in Caucasian individuals. While some *XPO5* SNPs appear to confer risk in certain cancers, these associations seem to be SNP-specific and highly dependent on population characteristics. In a recent Turkish study, Ağbektaş et al. examined mir146a polymorphisms (rs2961920 and rs2910164) in gastric, colon, and rectal cancers and reported that SNP–disease associations may vary considerably according to population structure and allelic distribution imbalances [33]. Consistent with their findings, our cross-sectional analysis of *XPO5* rs11544382 also revealed no significant differences in genotype or allele frequencies between cases and controls. Family history is a recognized risk factor for CC, with some studies reporting that familial cases constitute up to 20% of the patient population. However, similar to Ağbektaş et al., our study did not identify a significant association between CC and family history. Factors such as the advanced age of patients, unrecorded causes of death among relatives in rural regions, limited documentation, and generational lifestyle differences may contribute to these discrepancies. The observation that smoking and alcohol consumption were significantly associated with CC in our study further suggests that environmental exposures may modulate genetic susceptibility. Thus, the absence of a significant association between rs11544382 and CC should not be interpreted as a negative finding but rather as a reflection of population-specific allele frequencies, the typically small effect sizes of non-coding regulatory variants, and the substantial impact of environmental modifiers. Despite the well established role of *XPO5* in miRNA biogenesis and its documented dysregulation across multiple cancer types, the lack of association observed for rs11544382 in the present study suggests that not all *XPO5* alterations contribute equally to carcinogenesis. Importantly, many cancer-related changes in *XPO5* expression appear to arise from somatic mechanisms, such as chromosomal amplification, epigenetic regulation, or transcriptional deregulation, rather than inherited germline variants. Therefore, the absence of an effect for rs11544382 may indicate that this SNP is either functionally neutral or that its impact is highly population specific. This distinction underscores the need to differentiate between somatic alterations affecting *XPO5* function and germline polymorphisms with limited or context dependent effects.

## 4. Materials and Methods

### 4.1. Study Population, Design, and Biosafety

This study was conducted at the Department of General Surgery, Faculty of Medicine, Cumhuriyet University Training and Research Hospital (Turkey) using a cross-sectional case and control design. A total of 120 unrelated individuals were enrolled, including 60 patients with histologically confirmed CC and 60 healthy controls. All participants provided written informed consent prior to inclusion, and the study protocol was approved by the institutional ethics committee in accordance with the Declaration of Helsinki and institutional biosafety regulations. Patients with CC were consecutively recruited from the general surgery department, and diagnoses were confirmed histopathologically by a senior pathologist based on biopsy or surgical specimens. Individuals with a previous history of malignancy, autoimmune or chronic inflammatory diseases, or those receiving ongoing systemic medication were excluded. Healthy controls were recruited from individuals presenting to the same department for routine evaluation who underwent colonoscopic and endoscopic examinations and showed no pathological findings suggestive of colorectal cancer, adenomatous polyps, inflammatory bowel disease, or other colorectal disorders. Controls also had no history of malignancy or chronic systemic disease. Controls were matched to cases with respect to age and gender. All participants were recruited from the same geographic region and shared a common ethnic background; all were born in Turkey and had been long-term residents of the region. Demographic and clinical data, including socio-demographic characteristics, smoking status, alcohol consumption, and family history of cancer, were collected using a structured questionnaire. Peripheral blood samples were obtained from all participants. Sample collection and processing were performed in compliance with universal precautions for handling human specimens, and all procedures were carried out within a certified Class II biological safety cabinet to ensure biosafety and prevent contamination.

### 4.2. Preparation of Solutions and Buffers

The lysis buffer (pH 7.5) contained 10 mM Tris-base, 320 mM sucrose, 1% Triton X-100, and 4 mM MgCl_2_·6H_2_O and was stored at 4 °C before use. The TE buffer (pH 7.5) containing 10 mM Tris-base and 1 mM EDTA was pH-adjusted with HCl and stored at 4 °C. The TEN buffer (pH 8.0) containing 400 mM NaCl, 10 mM Tris, 2 mM EDTA, and 10% SDS was supplemented with 0.5 mL of 70% ethanol and stored at 4 °C.

### 4.3. Blood Sample Collection

Peripheral blood samples (3–4 mL) were collected from both colon cancer patients and healthy controls prior to the initiation of any treatment. Blood samples were obtained using sterile sodium citrate–containing tubes and processed for genomic DNA extraction.

### 4.4. Genomic DNA Isolation

Genomic DNA was isolated from peripheral blood samples using the high-salt DNA extraction method. Briefly, 4 mL of whole blood collected in sodium citrate tubes was transferred to a 15 mL polypropylene tube, and 4 mL of erythrocyte lysis buffer was added. The samples were centrifuged at 2200 rpm for 15 min, and the supernatant was discarded. This washing step was repeated 4–5 times until the supernatant became clear. Following erythrocyte lysis, the remaining pellet was resuspended in 600 μL of TEN buffer. Subsequently, 40 μL of 10% sodium dodecyl sulfate (SDS) and 7 μL of Proteinase K (15 mg/mL) were added, and the mixture was gently mixed by inversion. The samples were transferred to 1.5 mL microcentrifuge tubes and incubated at 55 °C for 3.5 h in a heating block to ensure complete protein digestion. After incubation, 200 μL of saturated NaCl solution was added to each sample, followed by centrifugation at 2600 rpm for 15 min. The supernatant was carefully transferred to a new microcentrifuge tube and centrifuged again at 3300 rpm for 30 min. The resulting supernatant was transferred to a 15 mL polypropylene tube, and genomic DNA was precipitated by adding two volumes of absolute ethanol. The precipitated DNA was transferred to a 1.5 mL microcentrifuge tube containing 200 μL of 70% ethanol and centrifuged for 10 min. The ethanol was removed, and the tube was left open for 5–10 min to allow residual ethanol to evaporate. Finally, the DNA pellet was dissolved in 40 μL of TE buffer and incubated overnight at room temperature. Purified DNA samples were stored at −20 °C until further analysis [34].

### 4.5. Determination of DNA Quality and Quantity

The concentration and purity of isolated genomic DNA were assessed using a NanoDrop spectrophotometer (Green Bioresearch, Wilmington, DE, USA). Absorbance values at 260 nm and 280 nm were measured using nuclease-free distilled water as a blank. DNA purity was evaluated based on the A260/A280 ratio, with values close to 1.8 considered indicative of high-quality DNA. Samples with ratios below or above 1.8, suggesting protein contamination or RNA presence, respectively, were subjected to reisolation.

### 4.6. XPO5 Genotyping

The *XPO5* rs11544382 (A/G) polymorphism was analyzed using quantitative qPCR with hydrolysis probes. Genotyping was performed with the SNPsig qPCR Genotyping Kit by Jena Bioscience (Frankfurt, Germany) using dual-labeled fluorescent probes (FAM/VIC). qPCR amplification conditions were as follows: initial denaturation at 95 °C for 2 min, followed by 15 cycles of denaturation at 95 °C for 2 min and extension at 60 °C for 60 s, and 40 cycles of denaturation at 95 °C for 15 s and extension at 68 °C for 60 s. Fluorescence data were collected through the FAM and VIC channels at the end of each cycle. Allelic discrimination was determined based on probe signals: FAM indicated the A allele (wild-type), while VIC indicated the G allele (mutant). Genotype distributions (AA, AG, GG) are presented in Table 1.

### 4.7. Ethical Considerations

The study protocol was reviewed and approved by the Sivas Cumhuriyet University Faculty of Medicine Ethics Committee (Approval Date: 17 April 2019; No: 2019-04/43). Written informed consent was obtained from all participants prior to enrollment, and a copy of the signed form was retained. The study posed minimal risk since participation involved only a brief questionnaire with non-sensitive data and blood samples collected during routine clinical tests, avoiding additional venipuncture. All samples were stored at −80 °C until the study objectives were completed. Confidentiality was strictly maintained: samples were coded with numerical identifiers, and personal information was stored on password-protected computers accessible only to authorized personnel. All data and sample exchanges were conducted using coded identifiers, ensuring that no personal or clinical information was linked to test results. Study staff were trained in safeguarding patient confidentiality.

### 4.8. Data Processing and Statistical Analysis

In this study, the association between CC and the *XPO5* rs11544382 polymorphism was investigated. Statistical analyses were performed using SPSS software, Version 23.0 (SPSS Inc., Chicago, IL, USA). The relationships between smoking status, alcohol consumption, and family history of cancer were examined. Genotype-associated odds ratios (ORs) with corresponding 95% confidence intervals (CIs) and *p*-values were estimated using unconditional logistic regression models. Differences in demographic characteristics between the cases and controls were evaluated using Student’s *t*-test. Pearson’s χ^2^ test or Fisher’s exact test (two-sided) was applied to compare gender distribution, assess associations between genotypes and alleles and disease status, and test for deviation from Hardy–Weinberg equilibrium. In addition, logistic regression analyses were performed to evaluate potential interactions between age, gender, and genotypes. Multivariable logistic regression analysis was conducted to estimate adjusted odds ratios, with age, gender, smoking status, alcohol consumption, and family history included as covariates. A *p*-value of <0.05 was considered statistically significant. In this research, the significance level was set at α = 0.01, the Type II error at β = 0.10, and the statistical power at (1 − β) = 0.90. Using these parameters, the required sample size was calculated with PASS software, (version 11, Kaysville, UT, USA) and it was determined that each of the two groups should include 60 individuals (2 × 60 = 120) (*p* = 0.9044).

## 5. Conclusions

This cross-sectional study examined the potential association between the rs11544382 polymorphism and CC. Our case–control analysis did not identify a significant relationship between rs11544382 and CC susceptibility, suggesting that germline variation at this locus is unlikely to contribute meaningfully to CC risk. To clarify the potential role of *XPO5* in colorectal tumorigenesis, future studies with larger sample sizes and multicenter designs will be essential for generating more robust evidence regarding genetic and environmental risk determinants. Additionally, synthesizing data from multiple studies conducted in Türkiye may facilitate a more accurate characterization of population-specific risk profiles.

## 6. Limitations

This study has several limitations. The sample size (60 cases and 60 controls) offers limited statistical power, as post hoc analysis indicated insufficient sensitivity to detect moderate genetic effects. The rs11544382 polymorphism also showed low allelic diversity, with the GG genotype absent in both groups, further reducing the ability to perform robust genotype-based comparisons. Additionally, the single-center design may limit the generalizability of the findings. Larger, multicenter studies with expanded sample sizes are needed to validate and extend these results.

## Figures and Tables

**Table 1:** Demographic characteristics of CC patients and controls.

Variable	Controls *n* (%)	CC *n* (%)	*p* Value
**Sample size**	60	60	
**Gender**			
Males	49 (48.0)	53 (52.0)	*0.306*
Females	11 (61.1)	7 (38.9)	
**Age (year)**			
Range	48–90	40–85	
**Median (min–max)**			
Males	60 (46–85)	64 (34–76)	*0.237*
Females	60 (47–75)	71 (43–85)	*0.053*
**Smoking History**			
Smoker	34 (76.4)	14 (46.6)	***0.001*** *
Males	33 (67.3)	13 (43.3)	
Females	1 (9.1)	1 (3.3)	
**Alcohol Consumption**			
Yes	4 (8.2)	13 (43.3)	***0.018*** *
Males	4 (8.2)	10 (33.3)	
Females	0 (0.0)	3 (10.0)	
**Family history of cancer**	10 (48.6)	8 (26.6)	*0.609*

* Statistically significant *p* values (*p* < 0.05). Chi-square test was used for categorical variables. Mann–Whitney U test was used for continuous variables (median, min–max).

**Table 2:** Allelic Distribution of *XPO5* rs11544382 in CC and Control Groups.

Allele	Controls *n*:60 (%)	CC *n*:60 (%)	χ^2^	*p*	Crude OR (95% CI)
***A***	65 (54.1)	68 (56.6)	0.15	*0.696* ^a^	0.90 (0.53–1.55)
***G***	55 (45.9)	52 (43.4)			

Values are presented as odds ratios (ORs) with 95% confidence intervals (CIs), ^a^ Chi-square test with *n* (%).

**Table 3:** Genotype Distribution of *XPO5* rs11544382 and Crude OR in CC Patients and Controls.

Genotype (Rs11544382)	Controls (Expected)	CC (Expected)	Crude OR (95% CI)
***AA***	5 (17.6)	8 (19.2)	0.59 (0.18–1.92)
***AG***	55 (29.8)	52 (29.5)	
***GG***	0 (12.6)	0 (11.3)	

Hardy–Weinberg equilibrium was tested using an exact test (controls and cases separately; Fisher’s exact test).

**Table 4:** Multivariable Logistic Regression Analysis of Clinical and Genetic Risk Factors for CC.

Variable	Crude OR (95% CI)	Adjusted OR (95% CI)	*p* Value
Genotype	0.59 (0.18–1.92)	0.62 (0.19–2.01)	*0.41*
Smoking	**3.84 (1.72–8.55)**	**3.12 (1.25–7.76)**	***0.014*** *
Alcohol	**3.87 (1.18–12.67)**	**2.93 (1.03–9.24)**	***0.042*** *
Family history	0.76 (0.28–2.10)	0.49 (0.14–1.64)	*0.22*

Values are presented as odds ratios (ORs) with 95% confidence intervals (CIs). * Significance indicated by *p* < 0.05. Adjusted ORs were obtained from a multivariable logistic regression model adjusted for age, gender, smoking status, alcohol consumption, and family history. Statistically significant *p* values are indicated in bold.

## Data Availability

The original contributions presented in this study are included in the article. Further inquiries can be directed to the corresponding author.

## Abbreviations

The following abbreviations are used in this manuscript:

CC	Colon cancer
Cls	Confidence Intervals
CRC	Colorectal cancer
miRNA	microRNA
ORs	Odds ratios
qPCR	Real-time PCR
RISC	RNA-induced silencing complex
RNAPII	RNA polymerase II
rs11544382	Genotyping of *XPO5*
SPSS	SPSS software, Version 23.0
*XPO5*	Exportin-5
χ^2^	Chi-square
